# Integrated Microbiome and Metabolomics Reveal Mechanism of Saline–Alkali Stress in *Avena sativa* L.

**DOI:** 10.3390/metabo16100785

**Published:** 2026-10-16

**Authors:** Guoling Ren, Beibei Yang, Zitong Chen, Rixin Wang, Yajie Yin, Anqi Xia

**Affiliations:** College of Bioengineering, Daqing Normal University, Daqing 163712, China

**Keywords:** oat, microbial diversity, root metabolites, salt–alkali stress

## Abstract

Background/Objectives: This study subjected four-week-old oats (*Avena sativa* L. cv. Qinqiong and *Avena sativa* L. cv. Menglong) to saline–alkali stress for 6, 12, 24, and 48 h, and non-targeted metabolomics was used to analyse root metabolites under stress. Differential metabolites in oat roots after saline–alkali stress were primarily concentrated in substances such as carbohydrates, amino acids, acids, purines, and pyrimidines. Methods: In addition, 16S rRNA high-throughput sequencing was used to analyse changes in the rhizosphere microbial community structure of oats under saline–alkali stress. The abundance of some taxa, such as *Nocardioides*, *Bacillus*, and *Proteobacteria*, increased. Spearman’s correlation analysis was used to determine correlations between differential microorganisms and metabolites. Results: Oats can secrete carbohydrate substances into the rhizosphere to recruit strains such as *Saccharothrix*, *Kineococcus*, and *Streptomyces*; aliphatic and heterocyclic amino acid substances to recruit *Arthrobacter*, *Bacillus*, and *Nostoc*; and acid substances to recruit *Accumulibacter*, thereby alleviating saline–alkali stress. Conclusions: These findings are significant for further studies on saline–alkali resistance in oats and for exploring the potential of oats’ inherent saline–alkali resistance. They also provide basic data and theoretical and practical support for promoting the sustainable development of agriculture in saline–alkali soils through the utilisation of microorganisms and metabolites.

## 1. Introduction

Saline–alkali stress refers to the simultaneous presence of high concentrations of salts and relatively high alkalinity in the soil, which exerts a series of inhibitory and harmful effects on the growth, physiological, and ecological processes of plants. Saline–alkali stress is the second most common abiotic stress after drought [1]. The worldwide area of saline–alkali land is approximately 932 million hectares [2]. The harm of salt stress is primarily caused by NaCl, Na_2_SO_4_, etc., resulting in a decrease in the osmotic potential of plant cells, a weakening of crop photosynthesis, an imbalance of ions, and an increase in the level of proline in plants. By contrast, alkaline stress is induced by alkaline salts such as NaHCO_3_ and Na_2_CO_3_; the resulting high pH damages cell membrane integrity and reduces root vitality and photosynthetic capacity [3]. The intensification of saline–alkali stress causes crops to lose water, consequently leading to their death. Moreover, saline–alkali conditions degrade soil structure, alter soil physicochemical properties, and reduce the bioavailability of soil nutrients, further aggravating the limitation on crop productivity.

Plant rhizospheres release a variety of substances to regulate themselves or establish connections with the rhizosphere to resist adverse conditions [4]. Previous studies have shown that saline–alkali stress prompts plant roots to secrete sugars, organic acids, and phenolic acids to enhance their osmoregulation and antioxidant capabilities and that the secretion of compounds such as amino acids and lipids is involved in stress signal transduction [5]. In Hunan, *Echinochloa frumentacea* adjusts the concentration of its metabolites and strengthens or changes carbon assimilation, respiration, and signal transduction to improve its adaptability to saline–alkali stress [6]. Therefore, screening plant metabolites using metabolomic methods has theoretical and practical significance for studying how plants adapt to saline–alkali stress and resist adverse conditions through root exudates.

Microorganisms can directly or indirectly promote the growth and yield of plants and help plants relieve the pressure of saline–alkali stress [7]. *Parametarhizium gen. nov.* improves the growth of mung bean plants under saline–alkali stress by increasing osmoregulatory substances and enhancing antioxidant capacity [8]. *Klebsiella oxytoca* improves the photosynthetic efficiency of plants and the saline–alkali tolerance of maize seedlings by increasing the activity of rhizosphere soil enzymes [9]. In conclusion, the tolerance of plants to saline–alkali stress can be enhanced through the combined action of plants and microorganisms and the ability of plants to remediate saline–alkali soil can also be improved. Therefore, screening microorganisms using microbiomics plays an important role in studying how plants relieve the pressure of saline–alkali stress through microorganisms.

Oats are plants of the Gramineae family, long-day crops, and dual-purpose crops used for both food and feed. They possess characteristics such as high yield, strong saline–alkali resistance, tolerance to infertile soil, and strong adaptability. Oats respond to environmental stress via osmoregulation, peroxidation, and ion regulation. Oats exhibit a relatively strong ion-selective absorption ability under saline–alkali stress. The plants will absorb and accumulate a large amount of Na^+^, reduce the absorption of K^+^, and synthesise organic solutes for osmoregulation to adapt to saline–alkali stress [10,11]. Oats are saline–alkali-tolerant plants that can grow normally in soil with a pH of approximately 8.5. Moreover, planting oats can improve soil structure and increase surface coverage. Meanwhile, oats themselves can absorb a portion of salt ions and remove them through harvesting, thus reducing the pH and salt content of the soil and achieving soil improvement.

In this study, four-week-old seedlings of the salinity–alkali-sensitive oat cultivar (*Avena sativa* L. cv. Menglong) and the salinity–alkali-tolerant cultivar (*Avena sativa* L. cv. Qinqiong) were exposed to salinity–alkali stress via pot culture for 6, 12, 24, and 48 h. This work aimed to characterise the short-term mechanistic responses of oat roots under salinity–alkali stress. Metabolomics profiles small-molecule metabolites to reveal biochemical alterations, and a non-targeted strategy was adopted to broadly explore rhizosphere metabolite changes under saline–alkali stress without compound preselection. The differential metabolites in oats in response to saline–alkali stress were screened using non-targeted metabolomics. The effect of saline–alkali stress on the composition of the microbial community in the rhizosphere soil of oats was investigated using 16S rRNA high-throughput sequencing technology. The interactions between oats, soil, and rhizosphere microorganisms were thoroughly understood. A molecular regulatory network of oats in response to saline–alkali stress was constructed. The mechanisms of oat roots, rhizosphere soil microorganisms, and metabolites in response to saline–alkali stress were explored. Few studies have combined rhizosphere metabolome and microbiome to characterise the early dynamic responses of contrasting oat cultivars to saline–alkaline stress, which constitutes the novelty of this work.

## 2. Materials and Methods

### 2.1. Plant Materials and Saline–Alkali Treatments

The pot experiment was carried out in 2022, from May to July. The seeds of *Avena sativa* L. cv. Qinqiong (Q) and *Avena sativa* L. cv. Menglong (M) oats were sown under controlled indoor soil conditions, with a light intensity of 200–230 μmol/(m^−2^·s^−2^), a photoperiod of 16 h, and illumination of 7000 Lux. Plants were cultivated with a daytime temperature of 25 °C; daytime humidity was maintained at 50%, while nighttime conditions included a temperature of 20 °C, lasting for 8 h, with humidity at 65%. A potted-plant method was adopted in this study; the seeds of Qinqiong oats were sown in a 3:1 mixture of soil and sandy soil sourced from the farmland of Daqing Normal University, within a greenhouse environment. Its basic physicochemical properties were as follows: pH = 8.09 ± 0.53, organic matter 24.22 ± 2.14 g/kg, available N 76.20 ± 1.57 mg/kg, available P 10.18 ± 0.60 mg/kg, and available K 199.08 ± 1.53 mg/kg. The plants were irrigated with Hoagland’s solution every two days. The seedlings were grown at a nighttime temperature of 18 °C, a daytime temperature of 24 °C, a relative humidity of 60–80%, and a photoperiod of 14/10 h (from 06:00 to 20:00 during daylight). Four weeks post-sowing, the oats were subjected to treatment with 150 mmol·L^−1^ of saline–alkali solution (Na_2_CO_3_:NaHCO_3_ in a 1:1 ratio). The pH of the prepared solution was stabilised at 9.8. This experimental formulation simulated the local soda–saline–alkali environment. Compared with simple NaCl-induced salt stress, this treatment better reflects actual field conditions, making the experimental results more relevant to field applications. The rhizosphere soil of the oats was sampled at intervals of 6, 12, 24, and 48 h following the stress treatment. After being washed with clean water, the samples were immediately frozen in liquid nitrogen and stored at −80 °C until RNA extraction for metabolite and microbiome sequencing analysis.

### 2.2. Sample Collection

Oat rhizosphere soil sampling: Rhizosphere soil was collected using the shaking-off method. The entire oat root system and soil as a whole were excavated; the soil around the roots was removed, and only the soil on the roots was retained. A total of 10 g of soil was obtained from each treatment, and three biological replicates were obtained from each treatment. Each sample was mixed. Bulk (non-rhizosphere) soil sampling: In the barrels from which the oat rhizosphere soil samples were taken, the soil was collected 10 cm from the plant to a depth of about 20 cm and put into a sampling tube. A total of 10 g of soil was obtained from each treatment, and three biological replicates were obtained from each treatment. Each sample was mixed. Oat root sampling: Oat plants were excavated with a shovel, the soil was washed off with tap water, and root tips with a length of approximately 5 cm were taken. Three biological replicates were taken from each treatment. Each group contained three samples. The biological samples were pooled before further processing and analysis. Each biological replicate corresponded to genuinely independent experimental pot units. The whole experiment was completed in a single experimental batch under controlled greenhouse conditions. Pots of both cultivars and all stress-time treatments were fully randomised within the growth chamber and re-randomised periodically to minimise positional bias. All samples were first frozen in liquid N, transported to the laboratory and stored in a −80 °C ultralow-temperature freezer until measurement.

### 2.3. Analysis of Oat Phenotypes and Physiological Indicators

We measured the plant height and root length of seedlings after 0, 6, 12, 24, and 48 h of saline–alkali stress. Seedling fresh and dry weights were determined after 6, 12, 24, and 48 h of stress exposure. Meanwhile, 1.0 g of root tissue collected from each treatment group was rapidly ground into powder in liquid nitrogen. The activities of catalase (CAT), superoxide dismutase (SOD), and peroxidase (POD), as well as the content of hydrogen peroxide (H_2_O_2_), were quantified using commercial assay kits obtained from Tianheng Biotechnology Co., Ltd. (Xi’an, China). All determinations were performed strictly according to the manufacturer’s instructions. The activities of antioxidant enzymes were expressed on a dry weight (DW) basis of plant tissue.

### 2.4. Determination of Rhizosphere Soil Metabolites

To determine the rhizosphere soil metabolites, 20 μL of an internal standard (L-2-chlorophenylalanine, 0.06 mg·mL^−1^; methanol configuration) and 1 mL of methanol–water (*v*/*v*, 1:1) were added to a 500 mg soil sample. Two small steel balls were included, and the mixture was kept at −20 °C for 2 min to precool before being ground in a grinder at 60 Hz for 2 min. The homogenised sample was then transferred into a 15 mL centrifuge tube containing 1 mL of methanol–water (*v*/*v*, 1:1). If any residue remained on the transfer tube wall, the preceding operation was repeated once. Following this, centrifugation was performed for 10 min at 7700 rpm and 4 °C. Subsequently, 2.5 μL of the supernatant was transferred to a 5 μL centrifuge tube, lyophilised, and then reconstituted with 400 μL of methanol–water (*v*/*v*, 1:4). This solution was vortexed for 60 s, sonicated for 30 s, and centrifuged for another 10 min at 12,000 rpm and 4 °C. Then, 150 μL of the supernate was drawn up using a syringe and filtered through a 0.22 μm organic phase needle filter. Then, the solution was transferred to an LC sample bottle and stored at −80 °C. Metabolite detection was performed on an LC-MS system comprising an ACQUITY UPLC I-Class plus ultrahigh-performance liquid chromatography (Waters Corporation, Milford, MA, USA) coupled with a QE high-resolution mass spectrometer (Thermo Fisher Scientific, Bremen, Germany). The LC-MS analysis was conducted by Shanghai Majorbio Bio-pharm Technology Co., Ltd., Shanghai, China. The following are the chromatographic conditions: column temperature: 45 °C; column: ACQUITY UPLC HSS T3 (100 mm × 2.1 mm, 1.8 µm); flow rate: 0.35 mL·min^−1^; mobile phases: A—water (containing 0.1% formic acid) and B—acetonitrile (containing 0.1% formic acid); injection volume: 2 μL. The heater temperature was set at 400 °C, and the capillary temperature was 320 °C. The sheath gas flow rate was 40 arb, and the auxiliary gas flow rate was 10 arb. The electrospray voltage was −2800 V in negative ion mode and 3500 V in positive ion mode. The normalised collision energy for MS/MS was 20–40–60 V. The resolution for full-scan MS was 70,000, and the resolution for MS/MS was 17,500. Data-dependent acquisition (DDA) mode was applied for data collection. The mass-to-charge ratio (*m*/*z*) detection range was 70–1050. For mass spectrometry, the ion source utilised was electrospray ionisation, with sample mass spectrometry signals collected in both positive and negative ion scanning modes. DNA concentration and purity were determined using a NanoDrop spectrophotometer (Thermo Fisher Scientific, Wilmington, DE, USA), and the A260/A280 ratio was calculated to evaluate DNA quality.

### 2.5. High-Throughput Sequencing

The study targeted the V3–V4 region of the bacterial 16S rRNA gene for PCR amplification. The amplification was performed using the reverse primer 798R (5′-AGGGTATCTAATCCT-3′) and the forward primer 343F (5′-TACGGRAGGCAGCAG-3′). Primers were synthesised by Shanghai Majorbio Bio-pharm Technology Co., Ltd., Shanghai, China. The following are the PCR conditions: denaturation for 30 s at 95 °C, annealing for 30 s at 55 °C, and extension for 45 s at 72 °C for 25 cycles, followed by a final extension at 72 °C for 20 min. After amplification, the PCR products were analysed using gel electrophoresis with 2% agarose to assess the amplification efficiency. PCR experiments were performed with technical replicates and negative controls. High-throughput sequencing was conducted on the Illumina MiSeq platform (Illumina Inc., San Diego, CA, USA) by Shanghai Majorbio Bio-pharm Technology Co., Ltd., Shanghai, China. The primary steps included extracting total microbial DNA from the soil following the operational procedures of the E.Z.N.A. Soil DNA Kit (Omega Bio-tek, Inc., Norcross, GA, USA), performing PCR amplification, and subsequently carrying out purification, recovery, and quantitative mixing. Finally, sequencing was performed on the sequencer to obtain paired-end (PE) reads.

### 2.6. Microbes and Metabolomics Analysis

Variance analysis was conducted on the preprocessed metabolomic matrix dataset. Principal component analysis (PCA) and partial least squares discriminant analysis (PLS-DA) were performed using the R package “ropls” (version 1.6.2). Model stability was evaluated using seven-fold cross-validation, and the risk of overfitting was further assessed using permutation tests with 200 permutations. The PLS-DA model yielded *R*^2^*X* = 0.669, *R*^2^*Y* = 0.972, and *Q*^2^ = 0.910. Additionally, Student’s *t*-test was performed. Significantly differential metabolites were screened based on the *p*-value obtained from Student’s *t*-test and the variable importance in projection (VIP) values derived from the PLS-DA model. Metabolites with VIP > 1.0 and *p* < 0.05 were defined as significantly differential metabolites. Differential metabolites identified in pairwise group comparisons were summarised and mapped to biochemical pathways for metabolic enrichment and pathway analyses. Pathway database searches were performed against the Kyoto Encyclopedia of Genes and Genomes database. Metabolites were classified according to their biological functions and the pathways in which they participate. Enrichment analysis was used to evaluate whether a given set of differential metabolites was overrepresented within functional pathway nodes, shifting the analytical perspective from individual-metabolite annotation to set-based analysis. Fisher’s exact test, implemented using the Python SciPy package (version 1.0.0), was used to identify significantly enriched pathways.

The V3–V4 hypervariable region of the bacterial 16S rRNA gene was amplified using primers 338F/806R and sequenced on an Illumina MiSeq platform (2 × 300 bp PE). Raw microbial data were demultiplexed by removing barcode and primer sequences, and paired-end reads were assembled to obtain RawTags. The raw data were then quality-filtered using Trimmomatic (version 0.33), (Phred Q < 20 trimmed; reads < 50 bp discarded), and chimaeras were removed using UCHIME (version 4.2.40) against the GOLD database, yielding EffectiveTags. Across 15 samples, 979,248 raw reads were obtained (mean, 65,283 per sample); after filtering, 860,871 effective sequences were retained (87.9% retention; mean, 57,391 per sample; length range, 203–517 bp; mean length, 415 bp).

OTU clustering was performed in QIIME (version 1.7.0) at 97% sequence similarity. Taxonomic annotation was conducted in Mothur (version 1.39.5) against the SILVA SSU rRNA database (release 132), with a confidence threshold of 0.8–1.0, yielding classifications from the phylum to genus levels. Bar plots of the 10 most abundant bacterial taxa were generated, with sample groups on the x-axis and relative abundance on the y-axis. Representative OTU sequences were aligned using PyNAST against the Greengenes database for phylogenetic tree construction. Sequencing data were rarefied to the minimum sequencing depth (52,988 sequences per sample), and alpha-diversity and richness indices (observed OTUs, Chao1, Shannon, and Simpson) were calculated using QIIME 1.7.0. Differences in alpha-diversity among groups were assessed using one-way analysis of variance with Tukey’s Honestly Significant Difference or, for nonnormally distributed data, the Kruskal–Wallis test with Dunn’s correction. Beta-diversity was visualised using PCoA based on Bray–Curtis dissimilarity, and compositional differences were tested using Permutational Multivariate Analysis of Variance with 999 permutations. Differentially abundant taxa were identified using LEfSe (version 1.1.2) (LDA > 2.0, adjusted *p* < 0.05). Rarefaction curves were plotted using the vegan package in R (v2.15.3); heatmaps and phylogenetic trees were constructed in R, with hierarchical clustering performed using the UPGMA algorithm.

### 2.7. Statistical Analysis

Statistical analyses were performed using R software (version 3.3.1) and QIIME (v1.9.1). Data normality was assessed by the Shapiro–Wilk test. Group differences in alpha-diversity were assessed by one-way ANOVA implemented via the vegan package (v2.4.3) in R or QIIME. When the main effect was significant, the LSD post-hoc test was applied for pairwise comparisons between group means. The Kruskal–Wallis test was used for non-normally distributed data. A Pearson correlation analysis for soil physicochemical properties was conducted in SPSS 22.0. Statistical significance was set at *p* < 0.05.

## 3. Results

### 3.1. Oat Phenotypes and Physiological Indices

The present study analysed the physiological indices of salt–alkali-tolerant and salt–alkali-sensitive oat varieties under saline–alkali stress at 0 h (CK), 6 h, 12 h, 24 h, and 48 h. Plant height and root length of the salt-tolerant variety decreased significantly with prolonged stress. The salt-sensitive variety exhibited initial increases followed by decreases in plant height and root length. The root length at M-6 h showed the most significant difference, increasing by 28.1% compared to the CK. Dry weight and fresh weight of the salt-tolerant variety initially increased and then decreased with prolonged stress. Dry weight at Q-24 h displayed the most significant difference, rising by 76.7% compared to CK. The salt-sensitive variety demonstrated a trend of initial increase, subsequent decrease, and partial recovery in dry weight and fresh weight (Table 1).

**Figure 1:**
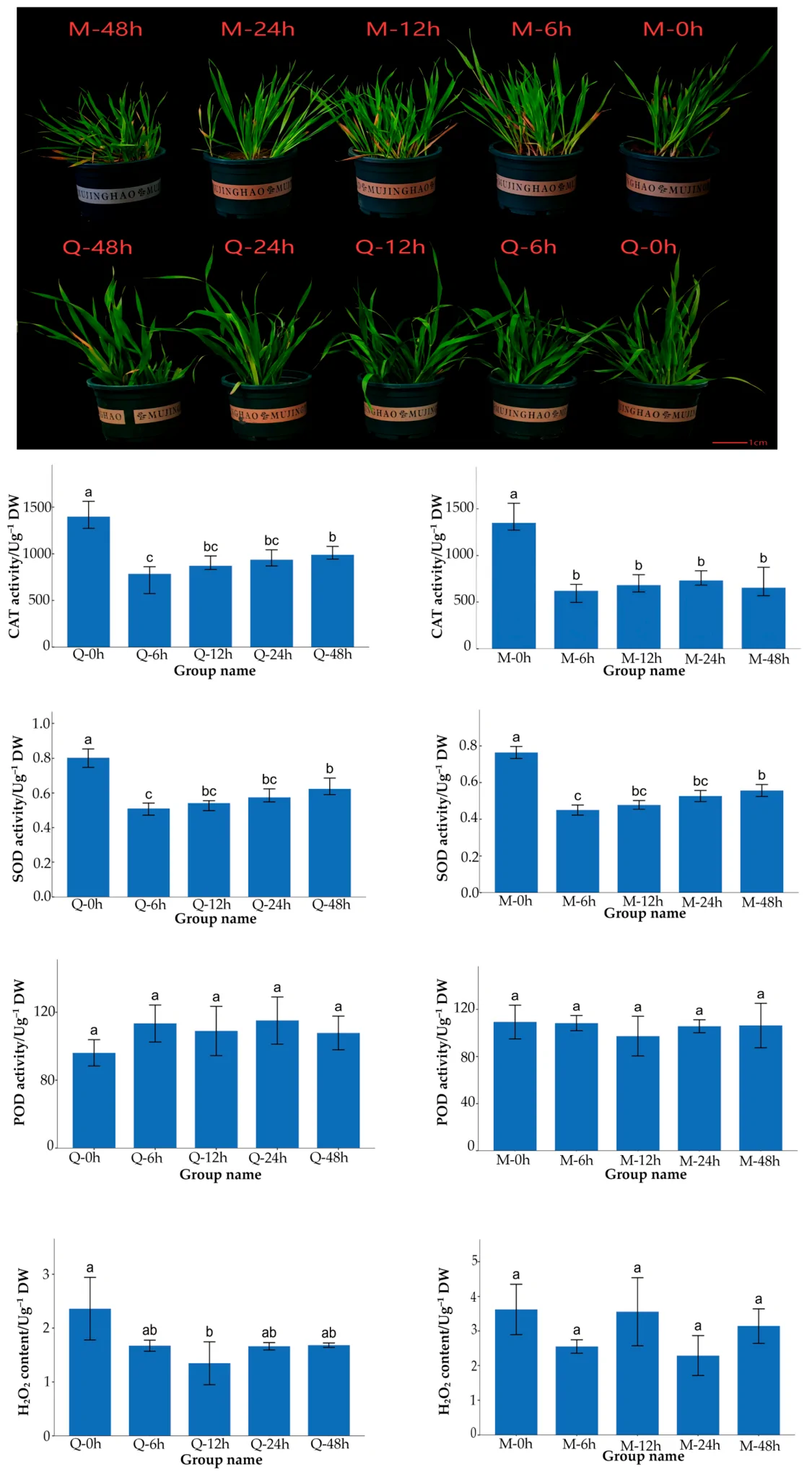
Root zone enzyme activity of oat under salt stress. Different lowercase letters denote significant differences between groups (*p* < 0.05). Q-0 h, Q-6 h, Q-12 h, Q-24 h, and Q-48 h indicate plants of saline–alkali-tolerant oat cv. Qinqiong under saline–alkali stress for 0, 6, 12, 24, and 48 h. M-0 h, M-6 h, M-12 h, M-24 h, and M-48 h indicate plants of saline–alkali-sensitive oat cv. Menglong under saline–alkali stress for 0, 6, 12, 24, and 48 h.

The present study investigated changes in CAT, SOD, and POD activities and H_2_O_2_ content in both oat varieties under saline–alkali stress. According to the results, CAT and SOD activities decreased significantly in both oat varieties after 6 h of stress, and then increased with prolonged stress. By contrast, POD activity maintained a relatively stable level with no significant difference across all stress time points in both varieties. In addition, H_2_O_2_ content decreased notably after 6 h of saline–alkali treatment, reached the minimum value at 12 h, and then rebounded gradually as stress duration extended. Both oat varieties showed similar dynamic patterns in POD activity and H_2_O_2_ accumulation under saline–alkali conditions (Figure 1).

### 3.2. Screening and Analysis of Differential Metabolites

**Figure 2:**
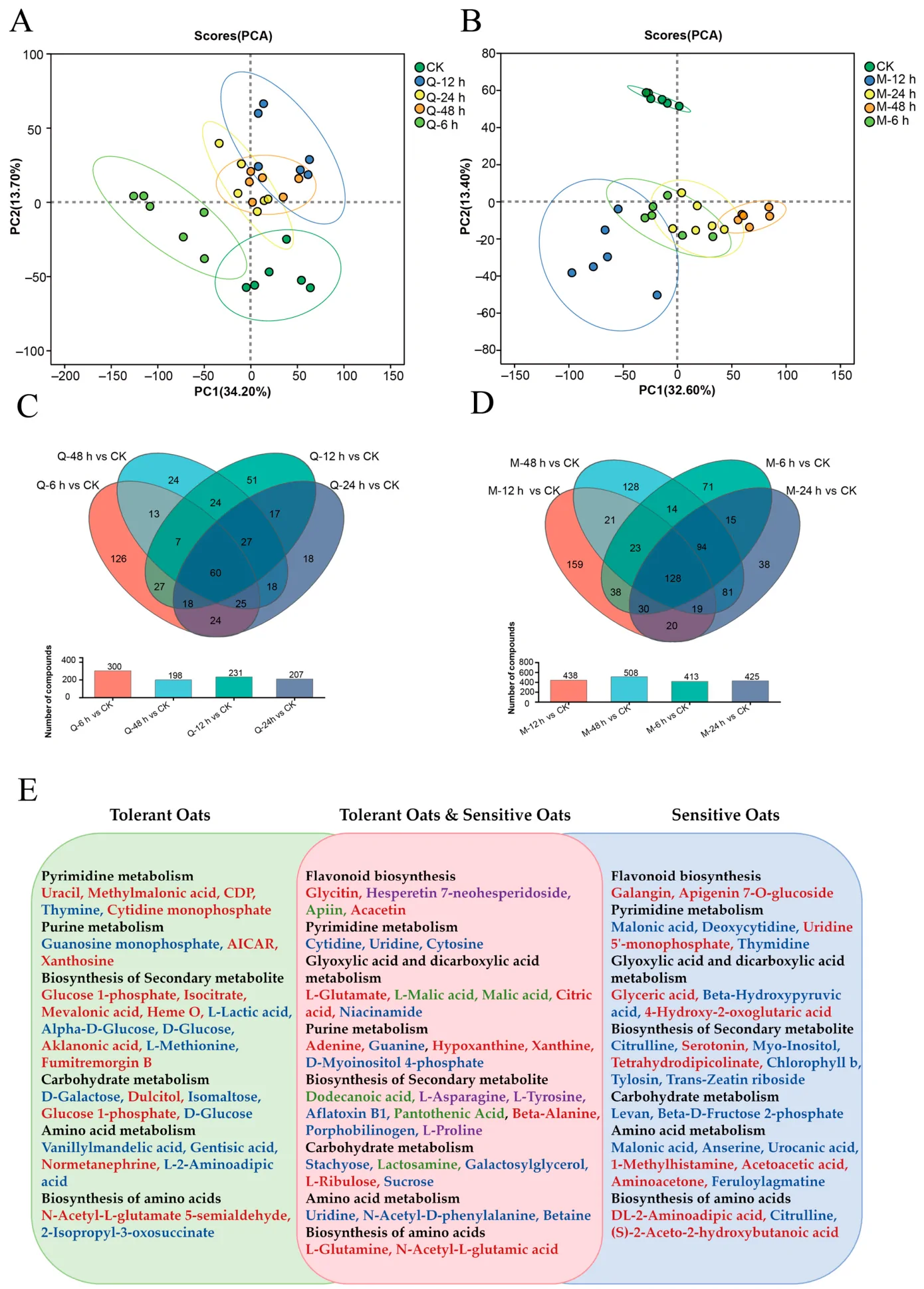
Differential metabolite diagrams of oats under saline–alkali stress. (**A**,**B**) PCA diagrams of rhizosphere metabolites of tolerant and sensitive oats. (**C**,**D**) Venn diagrams of rhizosphere metabolites of tolerant and sensitive oats. (**E**) Tolerant-type metabolite diagram of tolerant and sensitive oats. [Note: red indicates upregulation; blue indicates downregulation; purple indicates upregulation in sensitive oats and downregulation in tolerant oats; green indicates upregulation in tolerant oats and downregulation in sensitive oats.

The principal component analysis of the metabolites in the rhizosphere of oats showed that the sample group of the tolerant oat variety under 6 h saline–alkali stress treatment had a relatively large difference from the other four groups, and the sample group of the sensitive oat variety under 12 h saline–alkali stress treatment had a relatively large difference from the other four groups (Figure 2A,B), indicating that the metabolites in the rhizosphere of oats had an obvious response to saline–alkali stress during these two periods. Analysis of the Venn diagram of metabolites revealed that in the rhizosphere of the tolerant oat variety, the number of differential metabolites was up to 300 under 6 h of saline–alkali stress, primarily including malic acid, shikimic acid, maltotriose, phenylpyruvic acid, and deoxyinosine. In the rhizosphere of the sensitive oat variety, the number of differential metabolites was the largest, reaching 508, under 12 h saline–alkali stress, including sucrose, niacinamide, L-glutamine and sucrose (Figure 2C,D). There were 560 common metabolites between the tolerant and sensitive oat varieties under saline–alkali stress, such as cytidine, adenine, dodecanoic acid, stachyose, uridine and L-glutamine, which were primarily concentrated in flavonoid biosynthesis, pyrimidine metabolism, glyoxylate and malonate metabolism, purine metabolism, biosynthesis of secondary metabolites, sugar metabolism, amino acid metabolism, and biosynthesis of amino acids (Figure 2E).

### 3.3. VIP Values of Metabolites and KEGG Enrichment Analysis

**Figure 3:**
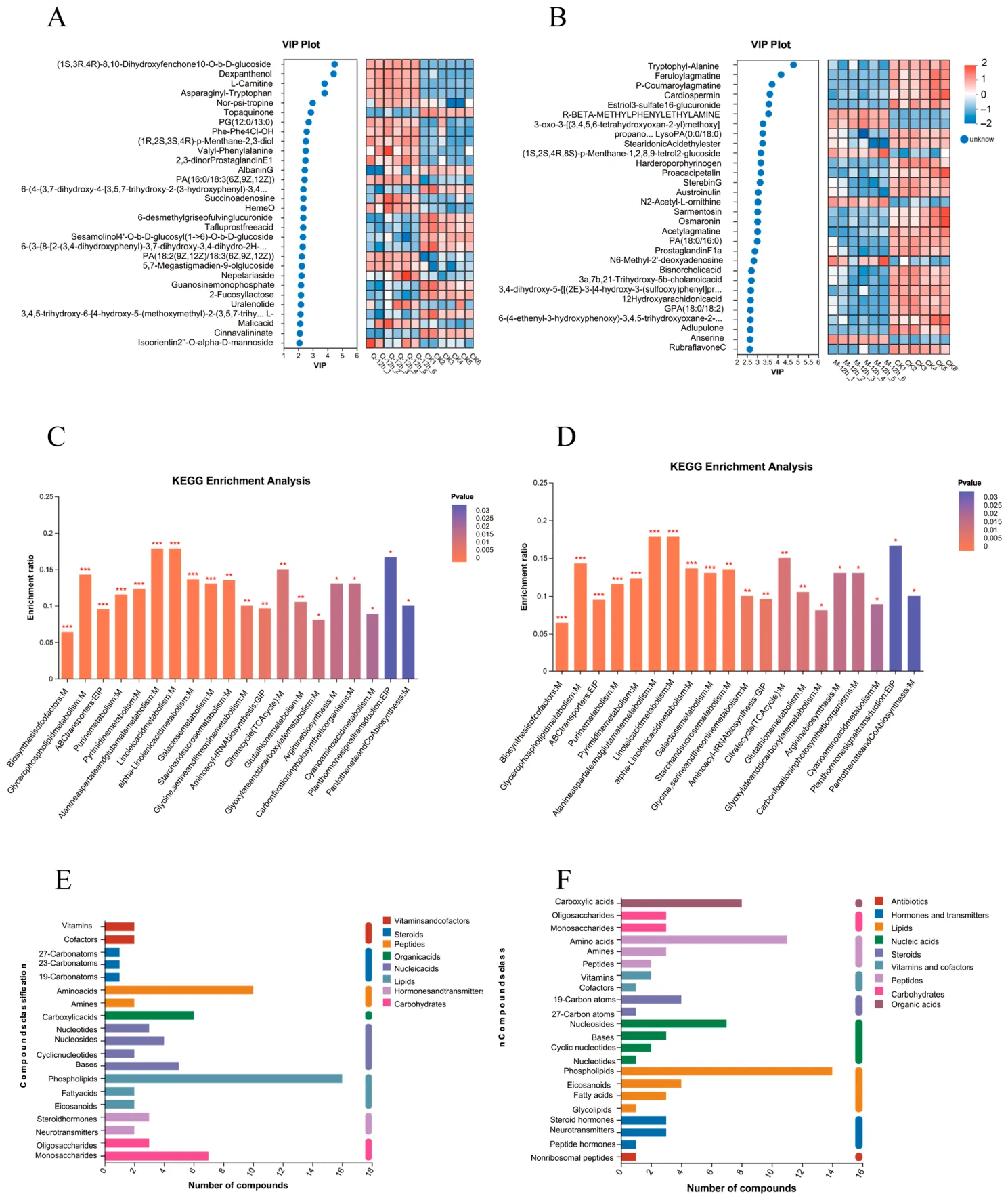
KEGG enrichment analysis of metabolites. (**A**,**B**) Heat map of VIP values of rhizosphere metabolites of tolerant and sensitive oats. (**C**,**D**) KEGG pathway enrichment map of tolerant and sensitive oat metabolites. (**E**,**F**) Tolerant and sensitive oat metabolite KEGG compound classification chart. * *p* < 0.05, ** *p* < 0.01, *** *p* < 0.001.

The top 50 differentially expressed metabolites were identified based on Variance Importance in Projection (VIP) values (VIP > 1). In the tolerant variety, the differential metabolites included methylcytosine, cytidine, D-maltose, L-malic acid, and vitamin K3. In the sensitive oat variety, the differential metabolites included N-acetylornithine and deoxyadenosine (Figure 3A,B). For the KEGG pathway enrichment analysis of oat metabolites, the pathways with a metabolite *p* value < 0.0005 were screened. It was found that the sensitive oat variety was involved in a total of eight metabolite pathways, including the biosynthesis of arginine, β-alanine metabolism, arginine metabolism, purine metabolism, etc. The tolerant oat variety was involved in nine metabolic pathways, including purine, pyrimidine, aspartate, glutamate, linoleic acid, and galactose metabolisms. Under saline–alkali stress, the differentially secreted metabolites of the two oat varieties were primarily enriched in sugar and amino acid biosynthesis and purine metabolism (Figure 3C,D). According to the KEGG compound classification diagram, in the tolerant oat variety, the top five KEGG compound classifications were phospholipids, amino acids, carboxylic acids, steroid hormones and carbohydrates. The top five KEGG compounds for sensitive oat varieties were phospholipids, monosaccharides, amino acids, vitamins, and carboxylic acids. (Figure 3E,F).

### 3.4. Structure and Difference Analysis of the Rhizosphere Soil Microbial Community

**Figure 4:**
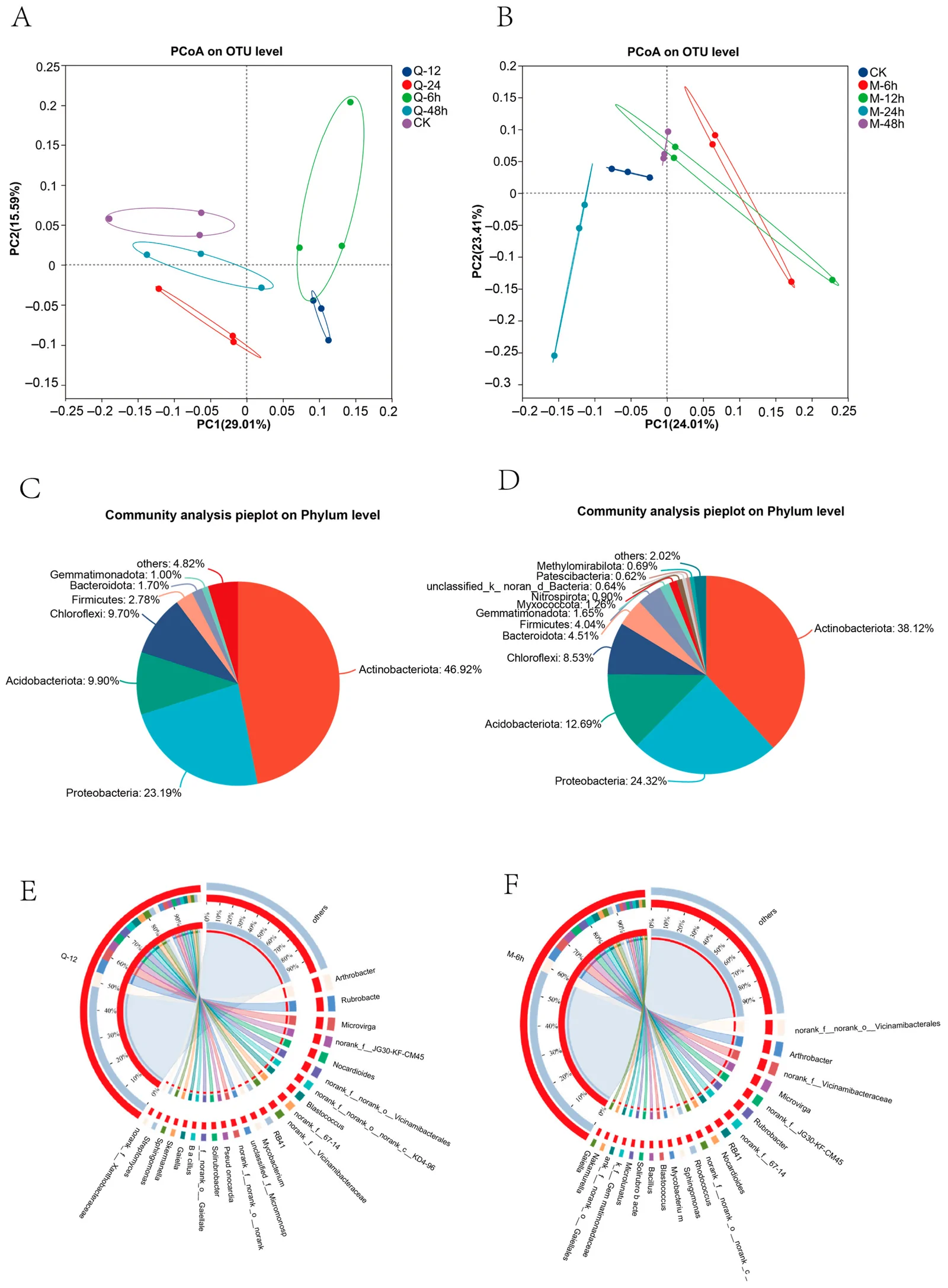
Structure and differential analysis of the rhizosphere soil microbial community. (**A**,**B**) Principal component analysis diagram of rhizosphere soil microbial community of tolerant and sensitive oats. (**C**,**D**) Tolerant and sensitive oat rhizosphere species community composition pie chart. (**E**,**F**) Tolerant and sensitive oat rhizosphere microorganism Circos diagram.

Principal component analysis (PCoA) showed that the sample group of the tolerant oat variety under the 24 h saline–alkali stress treatment had a relatively large difference from the other four groups, and the sample group of the sensitive oat variety under the 24 h saline–alkali stress treatment had a relatively large difference from the other four groups (Figure 4A,B).

At the phylum level, *Actinobacteriota* had the highest proportion in both tolerant and sensitive oat varieties, accounting for 46.92% and 38.12%, respectively, followed by *Proteobacteria*, *Acidobacteriota*, *Chloroflexi* and *Firmicutes*. After the tolerant oat variety was treated with 12 h saline–alkali stress, compared to the control group, the relative abundances of *Actinobacteriota*, *Chloroflexi*, *Acidobacteriota* and *Proteobacteria* were upregulated by 29.12%, 18.1%, 7.1%, and 5.2%, respectively. After treatment with 6 h saline–alkali stress, compared with the control group, the relative abundances of *Proteobacteria*, *Chloroflexi*, *Acidobacteriota* and *Actinobacteriota* were upregulated by 25.7%, 13.5%, 4.52%, and 3.84%, respectively (Figure 4C,D). At the genus level, the common dominant bacterial species in the rhizosphere of both the tolerant and sensitive oat varieties included *Arthrobacter*, *Microvirga*, *Vicinamibacterales*, etc., among which *Arthrobacter* had the highest proportion. The dominant bacterial species in the rhizosphere of the tolerant oat variety included *Rubrobacter*, *Blastococcus* and *Nocardioides*, whereas the dominant bacterial species in the rhizosphere of the sensitive oat variety included *Vicinamibacteraceae*, *Nocardioides* and *Sphingomonas* (Figure 4E,F).

### 3.5. Correlation Between Rhizosphere Microorganisms and Oat Metabolites Under Saline–Alkali Stress

**Figure 5:**
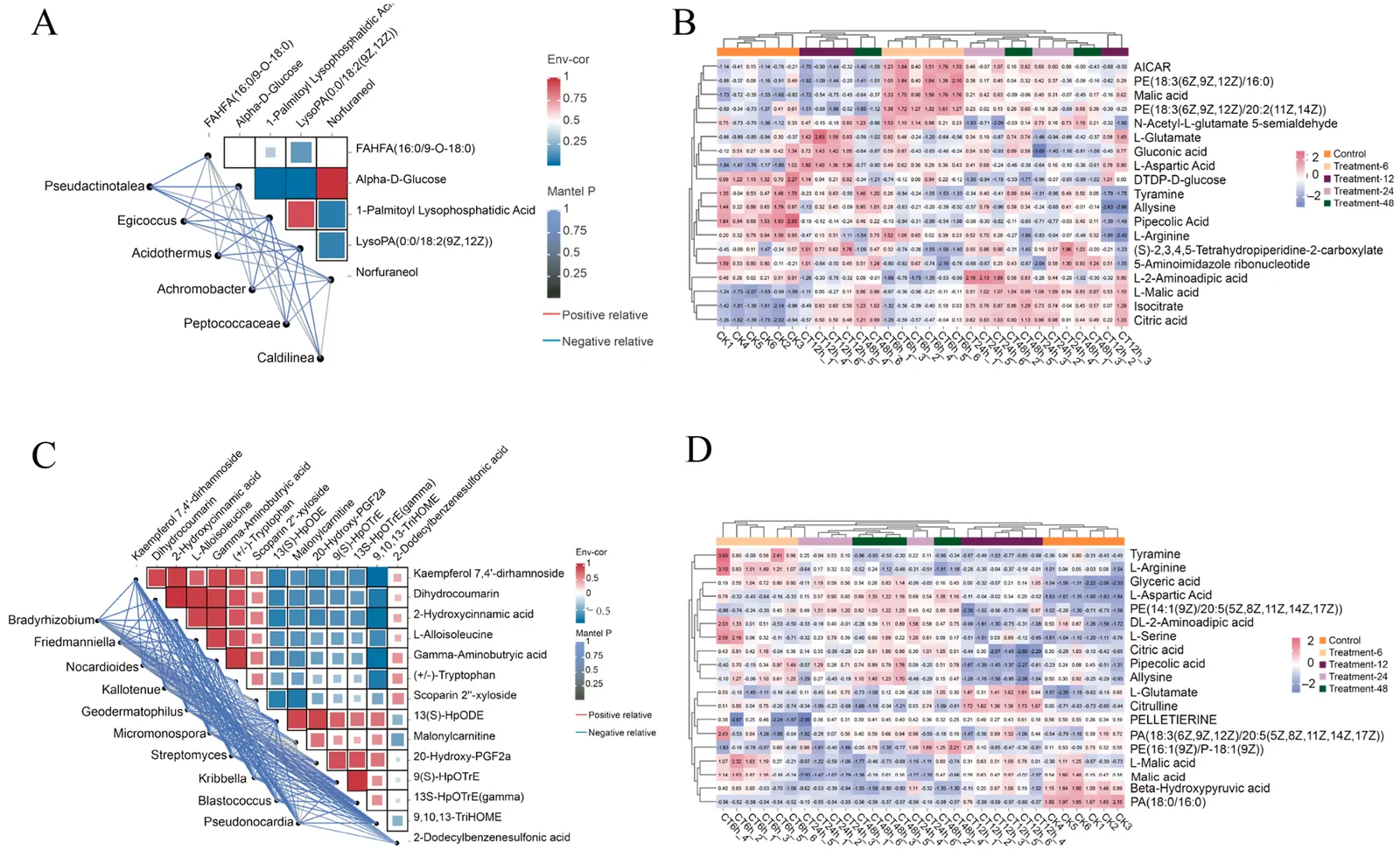
Correlation analysis between root metabolites and microorganisms. (**A**,**B**) Sensitive oats. (**C**,**D**) Tolerant oats.

As shown in Figure 5, the sample correlation analysis table was screened and metabolites with good correlations with microorganisms were grouped for analysis. In the rhizosphere of the sensitive oat variety, AICAR and DTDP-D-glucose presented significant positive correlations with *Pseudonocardia*, *Gaiella*, etc. Malic acid, L-glutamate, gluconic acid, and citric acid were negatively correlated with *Accumulibacter* abundance. Tyramine, allysine and L-arginine were positively correlated with *Arthrobacter* and *Mycobacterium*. In the rhizosphere of the tolerant oat variety, PELLETIERINE, malic acid, and beta-hydroxypyruvic acid were negatively correlated with *Achromobacter* and *Acidothermus*. L-malic acid, glyceric acid, and L-aspartic acid were negatively correlated with *Streptomyces*, *Nostoc* and *Methylomicrobium*. Beta-hydroxypyruvic acid and L-glutamate were positively correlated with *Neurinibacillus*.

Through correlation analysis between microorganisms and metabolites, as well as clustering analysis of metabolites, the metabolites were divided into four categories—carbohydrates, amino acids, purines, and pyrimidines.

## 4. Discussion

### 4.1. Carbohydrate Metabolic Substance Response to Saline–Alkali Stress

**Figure 6:**
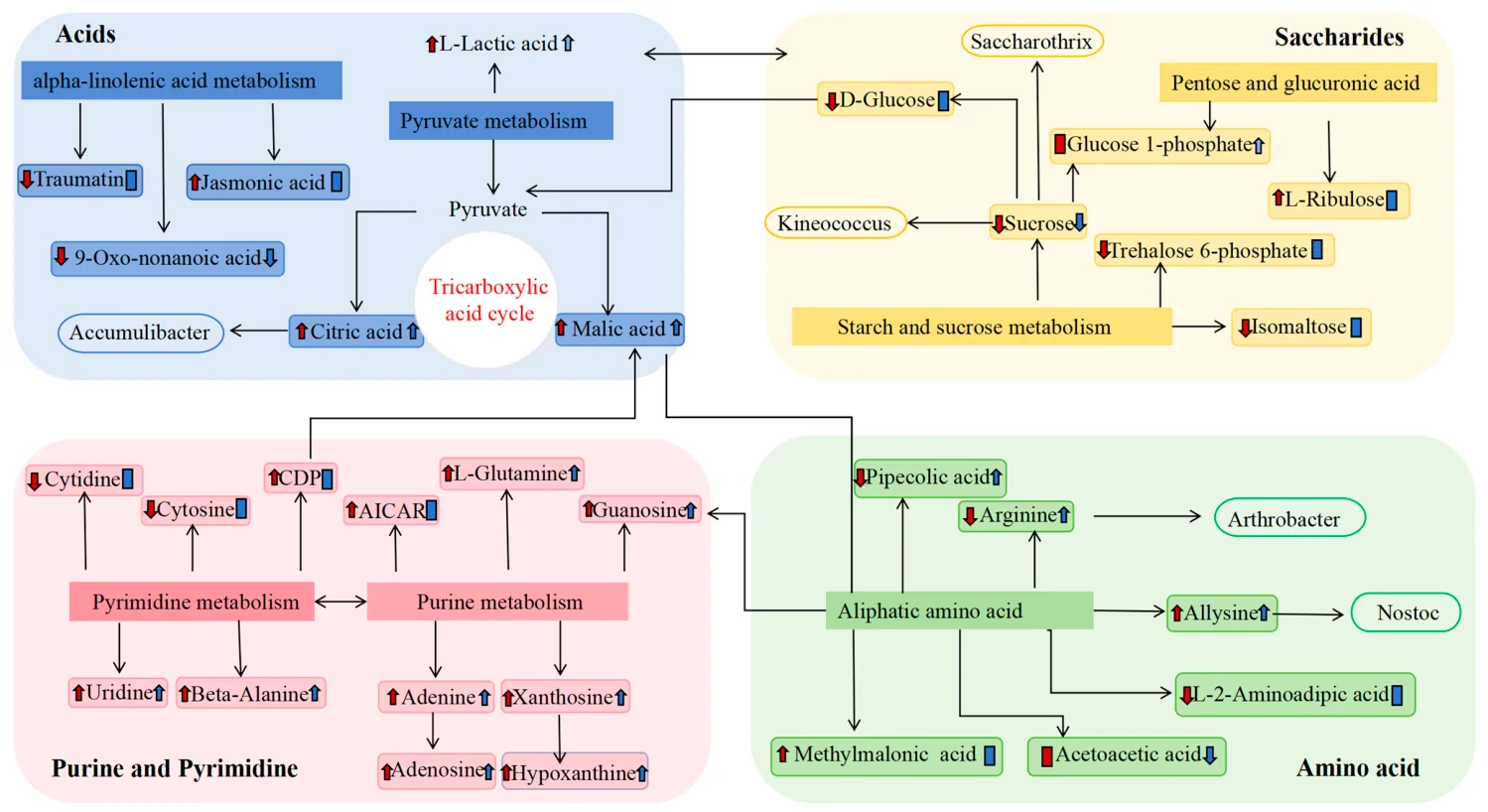
Graphical abstract. Red markers denote significantly upregulated metabolites, and blue markers denote significantly downregulated metabolites. Arrow-shaped markers represent changes in the sensitive oat variety, while square markers represent changes in the tolerant oat variety.

Carbohydrates enhance plant tolerance to various abiotic stresses. Studies have shown that plants can adapt to adverse conditions by regulating the synthesis and decomposition of sugars, thereby maintaining basic physiological activities [12]. As illustrated in Figure 6, carbohydrate metabolism constitutes a core module of the metabolite–microbe interactions in oat roots under saline–alkali stress.

Oat Vao-9 reduces damage caused by salt stress in plants through a mutual transformation between pentose and glucuronic acid [13]. Glucose-1-phosphate and L-ribulose, which are related to the mutual transformation of pentose and glucuronic acid, were only upregulated in the roots of tolerant oat varieties. Glucose-1-phosphate has antioxidant activity and promotes plant growth [14]. Pentose, an important carbon source, provides energy to plants through metabolic pathways to support their growth and physiological activities. Meanwhile, the metabolic products of pentose, such as amino acids and nucleotides, can also participate in the synthesis of important biomolecules, which are crucial for plant growth and response mechanisms [15,16].

Sucrose and its metabolites can act as osmoregulatory substances under stress conditions, helping plants maintain the osmotic balance inside cells and enhancing plant resistance to stress [17]. Trehalose-6-phosphate, a substance related to sucrose metabolism, was downregulated only in the rhizosphere of the tolerant oat varieties, D-maltose was upregulated only in the roots of the sensitive oat varieties, and sucrose was downregulated in the roots of both oat varieties. The relative abundances of *Saccharothrix* and *Kineococcus*, which were negatively correlated with sucrose content, were also upregulated. Studies have shown that some *Saccharothrix* species can decompose and transform carbohydrates into other substances through metabolism, thereby reducing the carbohydrate content in specific environments and playing an important role in enhancing plant abiotic stress resistance [18]. *Kineococcus* radiotolerans is a Gram-positive bacterium that is highly resistant to saline–alkali and osmotic stress and plays an important role in plant resistance to saline–alkali stress [19]. Maltose accumulation under salt and alkali stress helps maintain energy balance. Maltose is converted to glucose for energy metabolism and supports plant growth and survival under adverse conditions [20,21]. Trehalose-6-phosphate regulates the growth and development of plants and enhances plant responses to abiotic stress [22].

Galactose metabolism can provide the energy required for plants to maintain ion balance and synthesise antioxidant substances under saline–alkali stress, helping plants resist saline–alkali stress [23]. Here, D-galactose was downregulated only in the roots of the tolerant oat varieties, and stachyose was downregulated in the roots of both oat varieties. Stachyose, a functional oligosaccharide, enhances the resistance of sucrose-transporting plants to abiotic stresses [24]. The relative abundance of Streptomyces, which was negatively correlated with stachyose abundance, was also upregulated. Streptomyces not only optimises the structure of the plant bacterial community but also plays an important role in plant resistance to abiotic stresses [25]. The relative abundance of *Pseudonocardia*, which was positively correlated with stachyose content, was downregulated. In previous studies, *Pseudonocardia* has been related to cellulose degradation [26], which can cause damage to the plant cell wall. In this study, the decrease in the relative abundance of *Pseudonocardia* reduced adverse effects on plants.

### 4.2. Amino Acid Metabolic Substance Response to Saline–Alkali Stress

As shown in Figure 6, multiple root-derived amino acids are closely associated with the enrichment of specific rhizosphere bacterial taxa. Under saline–alkali stress, plants synthesise and accumulate amino acids to increase the osmotic pressure inside the cells, thereby alleviating the dehydration phenomenon under stress [27,28]. Moreover, sugars and amino acids complement each other. Sugar metabolism provides the energy and carbon sources required for amino acid synthesis that jointly participate in the adaptive responses of plants [29].

Studies have shown that an increase in the levels of the branched-chain amino acids leucine, isoleucine, and valine can regulate plant respiration, thereby enhancing tolerance to salt stress [30]. Methylmalonic acid (MMA), a substance related to metabolism, was upregulated only in the roots of tolerant oat varieties, whereas 2-methyl-3-oxopropanedioic acid and acetoacetic acid were upregulated only in sensitive oat varieties. Under salt stress, MMA may help plants cope with high-salt environments by regulating the expression of membrane transport proteins [31,32]. Acetoacetic acid can enhance the antioxidant capacity of cells, thereby protecting them from oxidative damage caused by salt [33].

The co-metabolism of arginine and proline has a synergistic effect and exhibits feedback regulation when plants cope with adverse conditions, such as saline–alkali conditions, thereby enhancing their stress resistance [34,35]. L-glutamate, a substance related to the metabolism of arginine and proline, was upregulated in the roots of both oat varieties, with the most obvious upregulation in the sensitive varieties, reaching 1.23 times. Under salt stress conditions, the application of L-glutamate can significantly improve plant growth, promote plant root development, and enhance plant tolerance to salt [36,37]. The relative abundance of Bacillus, which was positively correlated with proline content, was upregulated. Bacillus can enhance the tolerance of plants to adverse saline–alkali conditions by reducing the accumulation of sodium ions [38]. The relative abundance of *Arthrobacter*, which was positively correlated with arginine content, was upregulated. *Arthrobacter* has the potential to fix nitrogen and can colonise the rhizosphere of plants. The symbiotic relationship between Arthrobacter and plant roots can promote plant growth and enhance resistance to saline–alkali stress [39].

Lysine metabolism can enhance the antioxidant capacity of plants and reduce the damage to cells caused by reactive oxygen species (ROS) generated under saline–alkali stress, thereby enhancing the stress resistance of plants [40,41]. In this study, the relative abundance of Nostoc cyanobacteria, which was positively correlated with lysine content, was upregulated. Cyanobacteria can quickly synthesise small-molecule-compatible substances to resist adverse high-salt conditions from the outside world [42].

### 4.3. Acidic Substance Response to Saline–Alkali Stress

Under saline–alkali stress, an increase in organic acids not only helps maintain the acid–base balance of cells but also improves the photosynthetic characteristics of plants and promotes plant growth [43].

Figure 6 further displays the alterations in TCA-cycle-related organic acids and their associations with rhizosphere microbes under saline–alkali exposure. Under saline–alkali stress, the activity of the TCA cycle in plants increases significantly, thus releasing more energy to help plants accelerate physiological metabolic reactions [27]. In the present study, malic acid and citric acid, substances related to the TCA cycle, were upregulated in the roots of both oat varieties, and the upregulation was the highest in the roots of the tolerant oat variety, reaching 1.36 and 1.52 times, respectively. Under adverse conditions, the accumulation of malic acid can help plants maintain the pH balance inside cells and regulate osmotic pressure, thereby enhancing their stress resistance [44]. The accumulation of citric acid can regulate the metabolic pathways of plants and improve their growth in saline–alkali environments [40,41]. The relative abundance of Accumulibacter, which was positively correlated with citric acid content, was upregulated. Accumulibacter can improve the growth status of plants in saline–alkali environments by synthesising plant growth-regulatory substances (such as auxins and gibberellins, etc.). This interaction not only helps the stress resistance of plants but also promotes the healthy development of the soil microbial community [45].

In the biological metabolic pathway, pyruvic acid can be further transformed into acetyl-CoA and enter the tricarboxylic acid cycle to support the energy requirements and metabolic balance of cells [35]. Lactate, a substance related to pyruvic acid metabolism, was only upregulated in the roots of the tolerant oat varieties. Lactate regulates the stress resistance of plants through its interaction with ABA and plays a synergistic role in coping with environmental pressures [46].

### 4.4. Purine and Pyrimidine Metabolic Substances Response to Saline–Alkali Stress

As presented in Figure 6, purine and pyrimidine metabolism represents another critical metabolic module responding to saline–alkali stress in oat roots. Under salt stress, the adjustment of purine metabolism helps to maintain the physiological functions of plant roots. Its intermediate products can accumulate and further regulate the physiological processes of plants, thereby enhancing their adaptability to stress [47,48]. Xanthine, hypoxanthine, adenosine, and adenine, which are related to purine metabolism, showed the most obvious upregulation in the tolerant type, by 1.38, 1.41, 1.22, and 1.23 times, respectively. The metabolism of xanthine and hypoxanthine affects intracellular signal transduction pathways and regulates the response of plants to salt stress, enabling them to survive in high-salt environments [49]. Adenine is related to the metabolic pathway of zeatin biosynthesis. Adenine is converted into zeatin via the zeatin biosynthetic pathway. Zeatin is a member of the cytokinin family, which participates in plant growth and development by promoting cell proliferation and differentiation [50].

Pyrimidine metabolism is closely related to other metabolic pathways. Under adverse conditions, plants optimise their physiological responses by regulating the balance of these pathways, thereby enhancing their adaptability to adverse conditions [51,52]. Cytidine and L-glutamine, metabolites related to pyrimidine metabolism, are only downregulated in the roots of the tolerant oat variety; cytidine diphosphate and methylmalonic acid are only upregulated in the roots of the tolerant oat variety. Uridine and its derivatives play important roles in ATP synthesis. In high-salt environments, the metabolism of uridine may help plants improve their energy supply [53,54]. Under saline–alkali stress, cytidine metabolism enhances the content of nucleotides, amino acids, and organic acids. These metabolites play an important role in energy metabolism and biosynthesis in cells, helping plants resist saline–alkali stress [55].

### 4.5. Plant–Microbe–Metabolite Interactions in Cereal Crops

Integrated multi-omics studies on cereals have uncovered plant–microbe–metabolite crosstalk under abiotic stress. In wheat, rhizosphere metabolites govern the recruitment of beneficial bacterial consortia, and microbe–metabolite networks pinpoint taxa linked to disease resistance [56]. In rice, root exudates act as chemical signals recruiting rhizosphere bacteria, with drought stress reshaping metabolite–microbe correlations across genotypes [57]. For maize under saline–alkali stress, rhizosphere metabolic reprogramming remodels bacterial communities, and Mantel and Procrustes analyses validate robust metabolome–microbiome associations [58]. In line with these reports, our oat results indicate metabolite-mediated rhizosphere interactions are vital for saline–alkali tolerance.

## 5. Conclusions

Saline–alkali stress significantly reshapes the metabolite composition of oat roots, with malic acid, shikimic acid, L-glutamine, and sucrose exhibiting substantial changes, and markedly alters the structure of the rhizosphere microbial community, accompanied by significant increases in the relative abundances of *Accumulibacter*, *Arthrobacter*, *Bacillus*, and *Nostoc*. Among root-derived sugar metabolites, sucrose recruits *Saccharothrix* and *Kineococcus*, whereas stachyose recruits *Streptomyces*. These interactions optimise rhizosphere bacterial community assembly and contribute substantially to oat tolerance to abiotic stress. Among root-derived amino acids, proline recruits *Bacillus*, arginine recruits *Arthrobacter*, and lysine recruits *Nostoc*, collectively contributing to plant saline–alkali tolerance. Furthermore, root-secreted citric acid recruits *Accumulibacter*, which facilitates oat growth under saline–alkali conditions. Taken together, these metabolite–microbe interactions modulate rhizosphere bacterial community structure, support the healthy development of soil microbial communities, and enhance plant stress resistance, enabling oats to better adapt to saline–alkali stress.

## Figures and Tables

**Table 1:** Growth indices of oat under salt stress. Different lowercase letters within the same column indicate significant differences among treatments at *p* < 0.05 by LSD test.

	Plant Height/cm	Root Length/cm	Dry Weight/g	Fresh Weight/g
Q-CK	20.37 ± 2.08 a	9.03 ± 3.06 abcd	0.030 ± 0.005 bc	0.042 ± 0.006 bc
Q-6 h	18.87 ± 2.68 ab	9.87 ± 4.39 abc	0.036 ± 0.007 ab	0.057 ± 0.009 ab
Q-12 h	18.57 ± 0.97 abc	8.97 ± 1.12 abcd	0.023 ± 0.007 bcd	0.031 ± 0.008 bc
Q-24 h	16.37 ± 1.84 bc	7.33 ± 2.66 cd	0.053 ± 0.038 a	0.088 ± 0.071 a
Q-48 h	15.73 ± 0.38 c	5.17 ± 0.15 d	0.018 ± 0.009 bcd	0.027 ± 0.012 bc
M-CK	17.73 ± 2.62 abc	9.73 ± 0.49 abc	0.021 ± 0.012 bcd	0.027 ± 0.014 bc
M-6 h	18.80 ± 1.80 ab	12.47 ± 3.43 a	0.029 ± 0.009 bcd	0.048 ± 0.031 abc
M-12 h	17.50 ± 1.06 abc	11.83 ± 2.50 ab	0.009 ± 0.002 cd	0.013 ± 0.001 c
M-24 h	18.17 ± 0.84 abc	8.17 ± 0.84 bcd	0.007 ± 0.004 d	0.010 ± 0.004 c
M-48 h	16.93 ± 2.00 bc	9.93 ± 1.01 abc	0.014 ± 0.004 bcd	0.020 ± 0.005 bc

## Data Availability

The datasets generated for this study are available on request to the corresponding author.

## Abbreviations

The following abbreviations are used in this manuscript:

CAT	Catalase
MMA	Methylmalonic acid
SOD	Superoxide dismutase
SOP	Linear dichroism
VIP	Variance importance in projection
